# Digital health and modern technologies applied in patients with heart failure: Can we support patients’ psychosocial well-being?

**DOI:** 10.3389/fpsyg.2022.940088

**Published:** 2022-09-28

**Authors:** Izabella Uchmanowicz, Marta Wleklik, Marva Foster, Agnieszka Olchowska-Kotala, Ercole Vellone, Marta Kaluzna-Oleksy, Remigiusz Szczepanowski, Bartosz Uchmanowicz, Krzysztof Reczuch, Ewa Anita Jankowska

**Affiliations:** ^1^Department of Nursing and Obstetrics, Wroclaw Medical University, Wrocław, Poland; ^2^Institute of Heart Diseases, University Hospital, Wroclaw, Poland; ^3^Center for Healthcare Organization and Implementation Research (CHOIR), Boston VA Healthcare System, Boston, MA, United States; ^4^Department of General Internal Medicine, Boston University School of Medicine, Boston, MA, United States; ^5^Department of Medical Humanities and Social Science, Faculty of Medicine, Wroclaw Medical University, Wrocław, Poland; ^6^Department of Biomedicine and Prevention, University of Rome Tor Vergata, Rome, Italy; ^7^Department of Cardiology, University of Medical Sciences in Poznan, Poznan, Poland; ^8^Department of Computer Science and Systems Engineering, Wrocław University of Science and Technology, Wrocław, Poland; ^9^Department of Family and Pediatric Nursing, Faculty of Health Sciences, Wroclaw Medical University, Wroclaw, Poland; ^10^Institute of Heart Diseases, Faculty of Medicine, Wroclaw Medical University, Wroclaw, Poland

**Keywords:** digital health, heart failure, modern technologies, patient-centered outcomes, telemedicine

## Abstract

Despite advances in the treatment of heart failure (HF), the physical symptoms and stress of the disease continue to negatively impact patients’ health outcomes. Technology now offers promising ways to integrate personalized support from health care professionals *via* a variety of platforms. Digital health technology solutions using mobile devices or those that allow remote patient monitoring are potentially more cost effective and may replace in-person interaction. Notably, digital health methods may not only improve clinical outcomes but may also improve the psycho-social status of HF patients. Using digital health to address biopsychosocial variables, including elements of the person and their context is valuable when considering chronic illness and HF in particular, given the multiple, cross-level factors affecting chronic illness clinical management needed for HF self-care.

## Introduction

**Figure 1:**
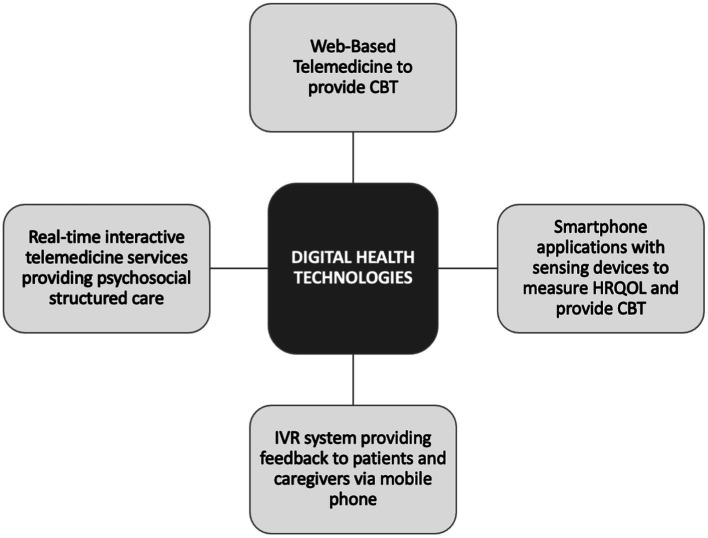
Scheme of main applications of digital health technologies in heart failure for biopsychosocial support.

The search for technological solutions to support HF patients in managing their physical and psychological well-being is important because self-management has been shown to decrease hospitalizations and improve survival (Meng et al., 2021). As digital technologies will play an increasing role in the management of HF patients, it is important to assess their impact on both physical and psychosocial support and identify those most effective to incorporate in daily clinical practice. This review discusses the use of digital health and modern technologies that can be used to support psychosocial well-being in patients with HF. The review will follow the pattern as seen in Figure 1.

The current global prevalence of heart failure (HF) is estimated at 64.3 million cases (Lippi and Sanchis-Gomar, 2020). This is expected to rise due to population growth, increasing numbers of older adults and improved survival after cardiovascular events (Obiegło et al., 2016, 2017). Research increasingly shows how selective and targeted use of digital health technologies—mobile health, remote patient monitoring, telemedicine and telehealth—can have several advantages in improving psychosocial support (De Witte et al., 2021; Farwati et al., 2021). Ideally, such strategies should be components of HF transition of care programs and include individualized interventions (Ong et al., 2016).

## Interactive voice response systems providing feedback

Interactive voice response, or IVR, allows patients to communicate with clinicians asynchronously using a mobile or landline telephone (Piette et al., 2013). Based on their responses, the patients can receive tailored feedback during the same call, and clinicians can receive alerts identifying patients who need additional assistance (Piette, 2000; Skolarus et al., 2017). Interactive voice response has also been used to facilitate telephone peer-to-peer support among older adults with HF (Heisler et al., 2007). Participants were paired with another patient who had HF who they contacted weekly using a toll-free IVR phone system. The study noted positive effects on participants’ HF social support and an improvement in depressive symptoms. In another IVR HF study (Zan et al., 2015), it was found that almost all participants reported feeling more connected to their health care team and their HF-related quality of life scores improved from baseline. Thus, indicating the efficacy of increased engagement and support with patients may alleviate loneliness and social isolation. The results from Clark et al. (2007) support the rationale for using IVR and telemonitoring as part of a comprehensive chronic HF management program. They show a high acceptability rate of 78% for such methods. As the study involved elderly patients, it can be considered that this vulnerable audience will be able to adapt to such solutions and accept them as part of the healthcare routine (Clark et al., 2007). The effectiveness of digital health solutions is dependent on the context in which they are applied. It has been suggested that interventions with more contact between clinicians and patients could be more effective (Chaudhry et al., 2010).

## Real-time interactive services

Real-time telemedicine, or interactive telemedicine, uses real-time interaction between patients and health professionals (Segato and Masella, 2017; Sasikala et al., 2018). Using real-time interactive services to address biopsychosocial variables, including elements of the person and their context is useful when considering chronic illness and HF in particular, given the multiple, cross-level factors affecting chronic illness clinical management and HF self-care (Holden et al., 2015a,b, 2017). In a study of a group-based HF telerehabilitation program found that participants liked the health benefits, access to care and social support (Hwang et al., 2017). The Better Effectiveness After Transition–Heart Failure (BEAT-HF) study found that telephone support improved the quality of life for patients 180 days after hospital discharge. The study offered: HF patient education before hospital discharge, regularly scheduled telephone coaching, and home telemonitoring of symptoms (Ong et al., 2016). Individuals participating in the intervention experienced improvements in their quality of life over the course of the study (Cordeiro et al., 2022). Research has shown that telephone contact to a trained nurse with access to a family physician, can prevent hospitalization in a quarter of HF patients (Kolasa et al., 2020). Therefore, patients with low social support or feelings of loneliness may therefore benefit from the support provided by telephone interventions (Blumenthal et al., 2019).

In a multisite randomized clinical trial (RCT) of the Collaborative Care to Alle*via*te Symptoms and Adjust to Illness (CASA) intervention, it was found that although HF-specific health status did not improve, secondary outcomes of depression and fatigue, both difficult symptoms to treat in HF, did improve (Bekelman et al., 2018). In yet another RCT comparing telemedicine versus a comprehensive outpatient management program, there was a demonstrated reduction in both anxiety and depression over the study period of 90 days (Pekmezaris et al., 2019). The Telemedical Interventional Management in Patients with Heart Failure (TIM-HF2) study suggests that a structured remote patient management intervention, can improve quality of life, while reducing the rate of lost days due to unplanned hospital admissions for cardiovascular causes and mortality from any cause (Koehler et al., 2018). Another study found that HF patients who received diet, medication and lifestyle teaching *via* real-time interactive services with nurses improved their mental health status, quality of life and an associated decrease in rehospitalization over a 1-year period (Mo et al., 2021).

## Smartphone applications

Mobile health (m-Health) is defined as medical and public health practice supported by mobile devices, such as smartphones, software apps on mobile devices, wireless sensors, etc. (WHO Global Observatory for eHealth, 2011; Sohn et al., 2020). The ubiquity of smartphones makes them a unique tool to use for providing psychosocial support. mHealth technology is increasingly being proposed for cardiovascular disease management (Lewis et al., 2016). Studies using mHealth interventions have shown to improve psychosocial functioning in those with noncardiac chronic medical conditions (Mlynarska et al., 2018; Sevilla-Cazes et al., 2018; Mansouri et al., 2019). This clear therapeutic potential indicates the possibility of mHealth as tool to improve the psychosocial status of those with HF. In a study using smartphones with wireless sensors, patient-reported outcome measures indicated improvement in fatigue, anxiety, depression, sleep disturbance, and social isolation (Sohn et al., 2020). The improvement persisted through the 180-day follow-up of the study. The HeartMan study, an RCT of HF patients, used a mHealth system that included a psychological support component. The psychological component included personalized messages based on cognitive behavioral therapy (CBT) and a weekly mindfulness games and exercises for relaxation and general psychological wellbeing. The findings suggest that psychological support has an important impact on patients, relieving their symptoms of depression, as well as their state and trait signs of anxiety (Toukhsati et al., 2015; Clays et al., 2021). This is in line with the growing evidence showing the ability of mindfulness to improve psychological well-being in chronic disease overall (Toback and Clark, 2017), and specifically in CHF (Löfvenmark et al., 2009). The results of HeartMan were similar to the home-based self-management psychosocial education intervention (HOM-HEMP) RCT (Jiang et al., 2021). In another study (Hägglund et al., 2019), using an mHealth system, participants reported that the system not only helped them better manage their heart failure but offered them psychological support, comfort, and a feeling of not being alone in their situation.

## Web-based cognitive behavioral therapy

Web-based or internet-based cognitive behavioral therapy (ICBT) is therapy provided through a computer or a mobile device (Lear et al., 2021). In ICBT, patients become active participants in their treatment and perform tasks to become aware of and to modify negative thoughts, emotions and unhelpful behaviors (Lundgren et al., 2016). A randomized clinical trial (RCT) of 230 participants compared the effect of an internet-based self-management and symptom monitoring program targeted (that included ICBT) to patients with HF and other chronic diseases (internet chronic disease management [CDM]) with usual care on hospitalizations over a 2-year period. The findings showed that self-management improved as well as social support as measured by the Medical Outcomes Study Social Support Scale (Sherbourne and Stewart, 1991). There was a significant change in favor of the internet CDM intervention in 2 of the 5 domains: emotional and informational support and overall support. In addition, fewer participants in the internet CDM vs. usual care group had at least 1 hospitalization and had a lower risk of time to first hospitalization.

An RCT investigating of 62 participants compared the effect of an ICBT program compared and an online moderated discussion forum in patients with HF (Lundgren et al., 2016). The study found a statistically significant improvement in depressive symptoms among patients in the ICBT group (Lundgren et al., 2016). In a follow-up qualitative study conducted with participants from this same RCT, findings revealed that participants perceived the support from the program as confirmative and motivating (Lundgren et al., 2018). Thus, the efficacy of using ICBT to improve psychosocial status remains a valid digital tool.

## Conclusion

In this review, we looked at several inter/intra-disciplinary digital health interventions that incorporated elements of psychosocial support. Among patients with HF, lack of emotional support has been shown to be a significant predictor of fatal and nonfatal cardiovascular outcomes within 1 year of hospital admission (Krumholz et al., 1998). Psychosocial support is therefore essential for maintaining physical and emotional well-being, supporting effective coping with HF symptoms, and improving patients’ life quality. Identifying the availability of social support should become an integral part of HF care, especially since in just over half of HF patients, having a partner does not translate into receiving high levels of support (Barutcu and Mert, 2013; Albert et al., 2015). Digital health technology now offers promising ways to integrate personalized support from health care professionals and improve the psychosocial status of HF patients (Van Spall et al., 2019) In conclusion, while there is no clear consensus on the type of digital technology and the optimal timing of its use in HF self-care, it is clear that these technologies have the ability to improve psychosocial wellbeing.

## Author contributions

IU, MW, MF, AO-K, EV, MK-O, RS, BU, KR, and EJ participated equally in this paper and were responsible for the paper conception, data collection, literature review as well as drafting and reviewing the manuscript. All authors contributed to the article and approved the submitted version.

## Funding

This review paper was supported by the Ministry of Health subventions according to the number of SUBZ.E250.22.095 from the IT Simple system of the Wroclaw Medical University in Poland.

## Conflict of interest

The authors declare that the research was conducted in the absence of any commercial or financial relationships that could be construed as a potential conflict of interest.

## Publisher’s note

All claims expressed in this article are solely those of the authors and do not necessarily represent those of their affiliated organizations, or those of the publisher, the editors and the reviewers. Any product that may be evaluated in this article, or claim that may be made by its manufacturer, is not guaranteed or endorsed by the publisher.

## Abbreviations

CBT: Cognitive behavioral therapy
HRQOL: Health-related quality of life
IVR: Interactive voice response
